# Human-Immune-System (HIS) humanized mouse model (DRAGA: HLA-A2.HLA-DR4.Rag1KO.IL-2RγcKO.NOD) for COVID-19

**DOI:** 10.1080/21645515.2022.2048622

**Published:** 2022-03-29

**Authors:** Teodor-D. Brumeanu, Pooja Vir, Ahmad Faisal Karim, Swagata Kar, Dalia Benetiene, Megan Lok, Jack Greenhouse, Tammy Putmon-Taylor, Christopher Kitajewski, Kevin K. Chung, Kathleen P. Pratt, Sofia A. Casares

**Affiliations:** a Department of Medicine, Division of Immunology, Uniformed Services University of the Health Sciences, Bethesda, MD, USA; b Infectious Diseases Directorate, Naval Medical Research Center, Silver Spring, MD, USA; c Bioqual Inc, Rockville, MD, USA

**Keywords:** Human immune system, humanized mice, DRAGA mice, COVID-19, lung immunopathology, SARS-CoV-2, human antibodies, human lung-resident CD8 T cells

## Abstract

We report a Human Immune System (HIS)-humanized mouse model (“DRAGA”: HLA-A2.HLA-DR4.Rag1KO.IL-2 RγcKO.NOD) for COVID-19 research. DRAGA mice express transgenically HLA-class I and class-II molecules in the mouse thymus to promote human T cell development and human B cell Ig-class switching. When infused with human hematopoietic stem cells from cord blood reconstitute a functional human immune system, as well as human epi/endothelial cells in lung and upper respiratory airways expressing the human ACE2 receptor for SARS-CoV-2. The DRAGA mice were able to sustain SARS-CoV-2 infection for at least 25 days. Infected mice showed replicating virus in the lungs, deteriorating clinical condition, and human-like lung immunopathology including human lymphocyte infiltrates, microthrombi and pulmonary sequelae. Among the intra-alveolar and peri-bronchiolar lymphocyte infiltrates, human lung-resident (CD103^+^) CD8^+^ and CD4^+^ T cells were sequestered in epithelial (CD326^+^) lung niches and secreted granzyme B and perforin, suggesting anti-viral cytotoxic activity. Infected mice also mounted human IgG antibody responses to SARS-CoV-2 viral proteins. Hence, HIS-DRAGA mice showed unique advantages as a surrogate *in vivo* human model for studying SARS-CoV-2 immunopathological mechanisms and testing the safety and efficacy of candidate vaccines and therapeutics.

## Introduction

Infection with Severe Acute Respiratory Syndrome-coronavirus-2 (SARS-CoV-2), the highly transmissible pathogen responsible for the ongoing pandemic coronavirus disease 2019 (COVID-19), results in outcomes from asymptomatic or mild disease to severe pneumonia and acute respiratory distress syndrome in the human population.^1–4^ So far, the World Health Organization (WHO) reported over 447 million established cases of COVID-19 and 6 million deaths.^5^ Many severe COVID-19 patients experience a hyper-inflammatory response (“cytokine storm”) combined with dysregulated coagulation.^6–11^ Both SARS-CoV-2 and the related SARS-CoV-1 coronavirus enter cells following engagement of their surface spike (S) protein with human angiotensin-converting enzyme 2 (hACE2)^12,13^ expressed on multiple epi/endothelial tissues and vasculature, including lungs, liver, colon, esophagus, small intestine, duodenum, kidney, brain, and tongue.^14^

More than 180 COVID-19 vaccine candidates are currently in development.^15^ Some vaccine preparations are based on chemically inactivated virions,^16,17^ or genetically attenuated live-virus based on “codon-pair bias de-optimization,”^18,19^ or “replication-incompetent vectors” using the various virus vectors.^20–24^ Older vaccination approaches refer as to recombinant proteins or virus-like particles expressed in insect cells, mammalian cells, yeast, and plants.^25–27^ Many of these vaccine platforms have entered clinical trials.^15^ Recently, newly developed RNA- and DNA-based preparations have been developed to deliver to the immune cells genetic information of the immunogenic antigens rather than protein antigens.^28–30^

International efforts are now aimed at developing pharmacotherapies to treat the severe SARS-CoV-2 infection.^31^ Some targeting drugs like Remdesivir, Hydroxychloroquine, and Lopinavir showed little to no effect on hospitalized COVID-19 patients.^32^ However, a large number of SARS-CoV-2 proteins such as Nsp13 (TBK1 and TBKBP1), Nsp15 (RNF41/Nrdp1), and Orf9b (TOMM70) and several host immune signaling proteins targeted by SARS-CoV-2 such as the IFN pathway, NF-κB pathway, and E3 ubiquitin ligases TRIM59 are being considered as potential drug targets and many entered clinical trials.^33^ Noteworthy, due to the complexity of SARS-CoV-2 immunopathology, the use of a single drug to treat COVID-19 maybe not a feasible approach.^34^ Among the candidate treatments, spike-specific monoclonal antibodies are promising immunotherapeutic agents.^35,36^ Clinically relevant animal models for SARS-CoV-2 infection are thus required for rapid testing of potential vaccines and immunotherapeutics that target the human immune system.

The SARS-CoV-2 S1 protein has significantly lower affinity for murine (m)ACE2 than for hACE2,^12,13^ and accordingly, wild-type mice are not susceptible to infection. Several murine strains transgenic for hACE2 expression driven by various promoters have been developed showing different tropisms, viral loads and pathologies,^37–40^ but they lack human anti-viral responses. Hence, a human immune system (HIS)-humanized animal model naturally expressing hACE2 and permissive for SARS-CoV-2 infection would be a highly appealing model to study mechanisms of viral entry and human-like anti-viral immune responses. HIS-humanized mouse strains has been already developed, but they do not mimic the human immune system with high fidelity due to poor engraftment of hematopoietic stem cells, or inefficient human cell expansion and homeostasis, insufficient numbers of reconstituted human T or B cells, sub-optimal B-cell development, lack of immunoglobulin class switching, lack of HLA class I and/or II T cell restriction, or development of GVHD.^41,42^ Furthermore, restrictions on the use of human fetal tissue for research (https://oir.nih.gov/sourcebook/ethical-conduct/special-research-considerations/policies-procedures-use-human-fetal-tissue-hft-research-purposes-intramural/policies) have focused attention on alternative donor cell sources, such as umbilical cord blood.

The HIS-humanized DRAGA (HLA-A2.HLA-DR4.Rag1KO.IL-2 RγcKO.NOD) mouse strain by virtue of expressing transgenically HLA class I/II molecules in the thymus promotes development of functional human T cells and is devoid of many of the above limitations.^43,44^ These mice are HIS-reconstituted after irradiation and infusion with HLA-matched CD34+ hematopoietic stem cells (HSC) from human umbilical cord blood. They lack the murine adaptive immune system while expressing a long-lived functional HIS. These mice respond vigorously by human specific T and B cell responses to infection or immunization with various pathogens including malaria protozoans, HIV, ZIKA, Scrub Typhus, and Influenza type A heterosubtypes.^45–51^ They also reconstitute various hematopoietic cell-derived human cells including endothelial cells (EDs) in the liver^45^and epithelial cells (ECs) in the lungs.^50^

Herein, we demonstrate that HIS-DRAGA mice naturally reconstitute human lung epi/endothelial cells expressing the hACE2 receptor for SARS-CoV-2 virus. Importantly, these mice sustained intranasal infection with SARS-CoV-2 for at least 25 days and developed dose-dependent, mild-to-severe human-like lung COVID-19 immunopathology. Infected HIS-DRAGA mice also developed human IgG antibodies to SARS-CoV-2 viral proteins and lung-resident human CD8 T cells expressing perforin and granzyme B. Hence, HIS-DRAGA mice showed unique advantages as a surrogate *in vivo* human model for studying SARS-CoV-2 immunopathology and for testing the safety and efficacy of candidate vaccines and therapeutics.

## Material and methods

### Humanized DRAGA mice and ethics statement

DRAGA mice express the HLA-A2.1 and HLA-DR0401 transgenes on a Rag1KO.IL2 RγcKO.NOD (NRG) background, and they have been described previously.^43,44^ De-identified umbilical cord bloods positive for HLA-A2.1 and HLA-DR0401 were commercially procured through the New York Blood Center (Long Island City, NY, USA (https://nybloodcenter.org/products-and-services/blood-products/research-products/). Mice were bred at the Veterinary Service Program at WRAIR/NMRC. Eight- to twelve-week-old mice were irradiated (350 rads) and injected intravenously with CD3^+^ T cell-depleted cord blood cells (EasySep Human CD3 Positive Selection Kit, cat# 18051, Stem Cell Technologies) containing approximately 10^5^ human CD34^+^ hematopoietic stem cells (HSC) as determined by FACS using a mouse anti-human CD34 antibody (BD Biosciences, cat# 550761). CD3 depletion of human T cells was required to avoid lethal (acute) graft-versus-host-reaction. Mice were infused with HSC from one of the 5 different cord blood donors in order to rule out the possibility of that infection and lung pathology could be related to a particular donor, gender, or HLA haplotype (Table 1). The procedures for assessing percentages of human T and B cells by FACS on the mononuclear FSC/SSC gate using human CD3 and human CD19 Abs (BD Biosciences, cat# 555339, # 555413) have been previously described.^43,44^ As documented in our previous studies, >90% of HSC-infused DRAGA mice reconstitute a human immune system by 3 to 4 months post-CD34^+^ HSC infusion.^43,44^ The human reconstitution status of DRAGA mice by percentages of human T and B cells in peripheral blood prior to SARS-CoV-2 infection is shown in Table 1 and Figure S1. Despite variability in the percentage of human B and T cells in different mice, statistical analysis using one-way analysis of variance (ANOVA) to test for differences over time for each type of cell indicated no significant difference among the six time points post-hHSC infusion for either B cells (F[5,21] = .4434, P = .8132) or T cells (F[5,21] = .6209, P = .6854). Based on a two-way ANOVA model comparing human B cells to human T cells over time, there was no overall difference between B and T cells averaging across time points (F[1,42] = 2.997, P = .0907). Furthermore, there was no significant cell type difference by time interaction (F[5,42] = .6681, P = .6497), indicating that differences between B and T cells did not vary significantly over time.

**Table 1.** Human immune parameters of HIS-DRAGA mice

			Peripheral blood					
Mouse*	Gender	Time post-infusion with human stem cells	% human B cells (CD19^+^)	% human T cells (CD3^+^)	SARS-CoV-2 challenge dose (pfu)	Euthanasia (days post-infection)	Cord blood HLA haplotype^	Cord blood gender
M#1	M	21 weeks	4.5	13.5	10^4^	1	A	M
F#1	F	21 weeks	48.6	9.3	10^4^	14	A	M
F#2	F	21 weeks	21	33.4	2.8x10^3^	14	A	M
F#3	F	24 weeks	16.1	37.8	1x10^3^	25	C	M
F#4	F	16 weeks	9.0	2.5	1x10^3^	25	B	F
F#5	F	24 weeks	6.0	23.8	1x10^3^	25	C	M
F#6	F	30 weeks	5.9	42.6	1x10^3^	25	A	M
F#7	F	16 weeks	23.6	5.0	1x10^3^	25	B	F
F#8	F	16 weeks	0.6	12.5	1x10^3^	25	B	F
F#9	F	16 weeks	21.5	8.0	1x10^3^	25	B	F
F#10	F	16 weeks	37.0	4.7	1x10^3^	25	B	F
M#2	M	30 weeks	8.3	9.3	1x10^3^	4	A	M
M#3	M	16 weeks	5.7	15.0	1x10^3^	4	B	F
M#4	M	16 weeks	2.0	51.1	1x10^3^	4	B	F
F#11	F	16 weeks	6.1	25.4	1x10^3^	4	B	F
F#12	F	24 weeks	7.0	8.8	1x10^3^	4	C	M
F#13	F	24 weeks	0.6	26.8	1x10^3^	4	C	M
a	M	20 weeks	18.9	28.3	—-	—-	D	F
b	M	20 weeks	0.9	41.4	—-	—-	D	F
c	F	24 weeks	52.5	17.5	—-	—-	D	F
d	M	24 weeks	7.4	16.6	—-	—-	D	F
e	M	24 weeks	1.0	22.1	—-	—-	D	F
f	M	24 weeks	1.2	44.7	—-	—-	D	F
g	M	24 weeks	17.6	38.3	—-	—-	D	F
h	F	20 weeks	35.2	5.0	—-	—-	E	M
i	M	26 weeks	4.9	26.1	—-	—-	E	M
j	M	26 weeks	21.9	30.7	—-	—-	E	M

Lungs from SARS-CoV-2 infected mice (M#2–4, F#11–13) that were euthanized at day 4 post-infection to estimate lung viral copies by RT-qPCR. Lungs from uninfected mice a-j were pooled to assess human ACE2 mRNA and protein expression.

^A: A02:01/A24:02/B13:02/B44:02/DR01:01/DR04:01.

^B: A01:01/A02:01/B08:01/B15:01/DR03:01/DR04:01.

^C: A02:01/A24:02/B44:02/B52:01/DR04:01/DR11:04.

^D: A02:01/A02:01/B18:01/B44:02/DR04:01/DR11:04.

^E: 02:01/A02:01/B08:01/B27:05/DR03:01/DR04:01.

The DRAGA mice were transferred to BIOQUAL Inc. for SARS-CoV-2 challenge experiments in their BSL-3 facility. All animal procedures reported herein were conducted under IACUC protocols approved by WRAIR/NMRC (#19-IDD-24) and BIOQUAL (#20-019P) in compliance with the Animal Welfare Act and in accordance with the principles set forth in the “Guide for the Care and Use of Laboratory Animals,” Institute of Laboratory Animals Resources, National Research Council, National Academy Press, 2011.

### RT-PCR detection of hACE2 mRNA in HIS-DRAGA mouse lungs

RNA was extracted using a Qiagen RNA extraction kit (Qiagen, cat# 74104) from lungs of HIS-DRAGA and control (non-HSC-infused DRAGA) mice. Human lung mRNA (Thermo Fisher, Scientific, cat#AM7968) served as a positive control. PCR primers specific for hACE2 were: forward, 5'-CAGGAAATGTTCAGAAAGCA-3' (exon#7) and reverse, 5'-TCTTAGCAGAAAAGGTTGTG-3' (exon #8) that were designed to bind to and amplify a 172 bp mRNA region of hACE2 (1055–1227 bp). The murine ACE2-specific primers used as internal housekeeping control were: forward: 5'-AGCAGATGGCCGGAAAGTTG-3' (exon#7), and reverse: 5'-TCTTAGCAGGAAAGGTTGCC-3' (exon#8) that were designed to bind to and amplify a 171 bp mRNA region of mACE2 (1338–1509 bp) (Eurofins). RT-PCR was performed using a One-step RT-PCR kit (Qiagen, cat# 210210) using 1 µg RNA template and 1.6 µM of each primer (20 µl reaction) using the following program: 50°C for 30 min, 95°C for 15 min followed by 45 cycles of 95°C for 30 s and 60°C for 30 s. Amplicons were run on a 3% agarose gel, purified from the gel, and nucleotide sequenced (Eurofins).

### RT-qPCR measurement of viral RNA copies in the lungs of SARS-CoV-2 infected HIS-DRAGA mice

Lungs RNA was extracted using RNA-STAT 60 extraction reagent (Tel-Test, Inc.) plus chloroform, precipitated, and re-suspended in RNAse-free water. Control RNA was isolated from SARS-CoV-2 viral stocks following the same procedure and quantified by OD260. These control stocks were serially diluted and OD260_nm_ values measured to generate a standard curve. RT-qPCR of the lung RNA was carried out using the following primers: 2019-nCoV_N1-F: 5'-GAC CCC AAA ATC AGC GAA AT-3'; 2019-nCoV_N1-R: 5'-TCT GGT TAC TGC CAG TTG AAT CTG-3'; and probe 2019-nCoV_N1-P: 5'-FAM-ACC CCG CAT TAC GTT TGG TGG ACC-BHQ1-3' (Integrated DNA Technologies) which were designed to bind to and amplify a conserved region of SARS-CoV-2 Nucleocapsid (N) RNA. Amplification was performed with an Applied Biosystems 7500 Sequence detector using the following program: 48°C for 30 min, 95°C for 10 min followed by 40 cycles of 95°C for 15 seconds, and 1 minat 55°C Reactions were carried out using a TaqMan RT-PCR kit (Meridian Bioscience, cat#BIO -78005) in 50 µL volume containing 5 µL of template, 2 µM of each primer and 2 µM of each probe. The number of viral RNA copies per mL was calculated by extrapolation from the standard curve, and values were then converted to the number of viral RNA copies per gram of lung tissue.

### Quantification of hACE2 protein in the lungs of HIS-DRAGA mice

Lungs from 10 non-infected HIS-DRAGA and 10 non-infected, non-HIS reconstituted DRAGA mice were homogenized in the presence of MPER mammalian protein extraction reagent (Fisher Scientific, cat# 78501) containing complete protease inhibitor cocktail tablets (Roche Diagnostics GmbH, cat# 1186153001) using tubes loaded with ceramic beads (MP Biologicals, cat# 6913100) in a Fast-prep homogenizer (MP Biologicals). Pooled lung homogenates from each group of mice were sonicated on ice in a Fisher Ultrasonicator for 10 cycles of 10 s each, the cellular debris was removed by centrifugation at 5,000 rpm, and the protein in the clear supernatant was quantified using a BCA reagent (Thermo Fisher Scientific, Cat# 23225). Nine mg of total lung protein extract from each group of mice and 2 mg of human lung total protein lysate (Zyagen, cat#HT-601) were then individually incubated with gentle shaking (300 strokes per min) in an Eppendorf thermomixer for 1 h at 37°C with 10 μg of the S1(RBD)-mFcγ2a protein (ACRO Biosystems, cat#S1N-C5257) followed by incubation with gentle shaking for 1 h at 37°C with 50 μl of rat anti-mouse IgG2a-magnetic microbeads (Miltenyi Biotech, cat#130-047-202). The total lysate from each sample was next passed over MACS magnetic columns (Miltenyi Biotech, cat#130-042-401), and the hACE2/S1(RBD)-mFcγ2a/rat anti-mouse IgG2a-magnetic beads were eluted according to the manufacturer’s instructions and concentrated to 52 μl each. The amounts of hACE2 protein in these immunoprecipitates were quantified using the highly sensitive hACE2 ELISA kit PicoKine^TM^ (Boster Biological Technology, cat#EK0997) per the manufacturer’s protocol. The recombinant hACE2 protein was serially diluted in the provided sample buffer. The human lung sample was diluted 1:100, and the DRAGA and HIS-DRAGA samples were diluted 1:50 each in the provided sample buffer. OD450 nm values were then read for duplicate samples (100 μl each) using a BioTEK microplate reader (BioTek Instruments). A hACE2 standard curve was constructed by applying a four-parameter logistic fit formula using the BioTEK Gen 5 software (BioTek Instruments Inc.). The sample OD450_nm_ mean values were then converted to hACE2 concentrations per manufacturer’s instructions.

### Immunoblot analysis of hACE2 protein in HIS-DRAGA lung lysates

Immunoblots were carried out using aliquots from the same immunoprecipitates used for ELISA quantification described above: immunoprecipitates obtained from (i) 2 mg human lung extract; (ii) 9 mg HIS-DRAGA mice lungs lysate; (iii) 9 mg DRAGA mice lungs lysate. 1 μl of each immunoprecipitate was added to wells of a 4–12% Bis-Tris gradient pre-cast gel (Thermo Fisher, Scientific, cat#NP0335PK2) and the samples were electrophoresed under denaturing conditions and electro-transferred onto a PVDF membrane. The membrane was blocked overnight at 4°C with gentle shaking in 5% nonfat milk plus 3% BSA in PBS, then incubated with a mouse monoclonal anti-human ACE2 (Abcam, cat#Ab89111) for 2 h at room temperature, and washed with PBS +.01% Tween 20. The membrane was further incubated with goat anti-mouse IgG-HRP (Santa Cruz Biotechnology, cat#Sc-2005, 1:3000) and SuperSignal^TM^ West Pico PLUS chemiluminescent substrate according to the manufacturer’s instructions (Thermo Fisher Scientific, cat# 34579). The chemiluminescent bands were imaged using a Fluorchem E Imaging System (Protein Simple).

### Infection of mice with SARS-CoV-2 virions

HIS-DRAGA mice were anesthesized with ketamine (35 mg/Kg body weigh) plus xylazine (5 mg/Kg body weight) injected intraperitoneally, and infected intranasally (i.n.) with SARS-CoV-2 strain USA-WA1/2020 (BEI Resources NR‐ 52281, batch # 70033175), which was provided to Bioqual, Inc. by the Centers for Diseases Control and Prevention (CDC). This virus strain was originally isolated from an oropharyngeal swab of a patient with a respiratory illness who had returned to Washington State, USA, from travel to China and developed COVID-19 in January 2020. Infection of HIS-DRAGA mice, harvesting of serum and organs, and all experiments requiring BSL-3 conditions were conducted in a BSL-3 laboratory at Bioqual, Inc. (Rockville, MD, USA). The SARS-COV-2 stock was expanded at Bioqual in Vero E6 cells, and the challenging virus was collected at day 5 of culture when the infection reached 90% cytopathic effect. The full viral genome sequence showed 100% identity with the parent virus sequence listed in GenBank (MN985325.1). A plaque-forming assay carried out with confluent layers of Vero E6 cells was used to determine the concentration of live virions, reported as plaque-forming units (pfu). HIS-DRAGA mice were infected i.n. with three different doses (10^4^, or 2.8x10^3^, or 10^3^ pfu/mouse) of SARS-COV-2 virus strain NR‐ 52281, batch # 70033175. The end point for euthanasia was 20% weight lost and severe clinical symptoms for 2–3 consecutive days, or if mice seemed moribund.

### ELISA measurement of serum antibody titers to SARS-CoV-2 viral proteins

Titers of human IgM and IgG serum antibodies (1/20 serum dilution) to the recombinant S1(RBD) viral protein from mice infected i.n. with SARS-CoV-2 virions (10^3^ pfu/mouse) were measured prior to infection and at 25 days post-infection (dpi) using SARS-CoV-2 human IgM and IgG ELISA kits according to the manufacturer’s instructions (Bethyl Laboratories, cats#E88–302, #E88–301). In addition, titers of human IgM and IgG serum antibodies in sera samples against a recombinant His-tagged super stable S trimeric protein and a recombinant His-tagged N protein (ACRO Biosystems, cats#SPN-C52H9, #NUN-C51H9) were determined using an in-house ELISA. Briefly, His-tagged S trimeric protein or His-tagged N protein, respectively, were coated on high-binding ELISA plates (Fisher Scientific, cat#07-200-721) at .2 μg/well/100 μL in carbonate buffer, pH 9.0 overnight at 4°C then blocked with PBS/1% BSA for 2 h at room temperature, washed with PBS/.05% Tween 20, and incubated at room temperature for 1 h with the sera samples diluted in PBS/1% BSA/.05% Tween 20. Bound human IgM and IgG antibodies against the His-S trimer protein were then revealed by adding anti-human IgM or IgG antibody-HRP conjugates (Bethyl Laboratories, cat#A80-100P, #A80-104P). Due to limited sera volumes, total human antibodies against the His-N protein were revealed by adding anti-human kappa plus lambda antibody-HRP conjugates (Bethyl Laboratories, cat#A80-115P, #A80-116P) to the His-N protein coated plates incubated with sera. The ELISA plates were then incubated with soluble TMB substrate (Bethyl Biolabs) for 15 min, and reactions were stopped by adding H_2_SO_4_ (.18 M, 100 μL/well). Plates were read in an ELISA reader at 450_nm_ and 570_nm_. OD450_nm_ values were corrected by subtracting the OD570_nm_ values. Standard deviations (±SD) for each serum sample in duplicate wells from two measurements were determined at 99% interval of confidence by SigmaPlot version 14 software. Human anti-S1 (RBD) antibody from the kit was used as a positive control for both S1 (RBD) and S1-trimer binding ELISA assays. The antibody titers against the N protein in serum from a non-infected mouse served as a negative control for the N-binding ELISA assays. Specificity controls for SARS-CoV-2 Abs were sera from HIS-DRAGA mice challenged with a sub-lethal dose of influenza PR8 virus at 21 days post-infection (Figure S5).^49,50^

### Histopathology of lungs from infected HIS-DRAGA mice

Lungs harvested from infected mice at the experimental end-points (14 dpi or 25 dpi) and trachea were either fixed in 10% formalin and embedded in paraffin blocks or prepared as frozen OCT blocks. 5 μm sections were stained with Hematoxylin/Eosin (H&E) or Masson’s trichrome reagent by Histoserv, Inc. (Germantown, MD, USA). Microscopic images were captured using an Olympus B×43 microscope (Shinjuku-ku, Tokyo, Japan).

### Immunofluorescence microscopy

Tissue sections (5 µm) from paraffin-embedded or frozen OCT cassettes from infected and non-infected HIS-DRAGA mice, and from non-infected, non-HIS reconstituted DRAGA mice were prepared at Histoserv, Inc. Thawed OCT-frozen tissue slides were rehydrated with PBS, and paraffin-embedded sections were de-paraffinized with xylene and rehydrated with graded concentrations of ethanol. Slides were then fixed, permeabilized with fixation/permeabilization buffer (Invitrogen, ‎Waltham, MA, USA), blocked with 3% BSA in PBS for 30 min at 37℃, and stained with fluorochrome-conjugated antibodies in PBS containing .01% Evans Blue at 37℃ for 50 min. To visualize hACE2, slides were probed with S1 (RBD)-mFcγ2a protein (10 µg/ml), washed with PBS, and then incubated with polyclonal goat anti-mouse IgG-FITC conjugate (Southern Biotech, cat#1013–02). Other antibodies to detect antigens of interest were: anti-human CD3-FITC (cat# 555339), anti-human CD4-PE (cat# 345769), anti-human CD8-PE (cat# 555635), anti-human CD45-FITC (cat# 347463), anti-human granzyme-B-PE (cat# 561142), anti-human CD103-FITC (cat# 550259) (all from BD Biosciences), anti-human CD326-PE (Miltenyi Biotech, cat#130-110-999), anti-human Perforin-PE (Biolegend, cat# 353304), anti-mouse CD61-PE (Invitrogen, cat#12-0611-82), anti-hACE2 antibody (Abcam, cat#Ab -89111), goat F(ab’)2 anti-mouse IgG1-PE conjugate (Southern Biotech, cat#1072–09), and goat F(ab’)2 IgG anti-mouse IgG2a (Southern Biotech, cat#1082–02). After staining, the slides were washed 3 times with PBS, air-dried, and mounted with Vectashield containing DAPI (Vector Laboratories, cat#H-1200-10). Immunofluorescent images were acquired with a Zeiss Axioscan Confocal microscope or Olympus B×43 microscope.

## Results

### HIS-DRAGA mice naturally reconstitute human lung epi/endothelia cells that bind to SARS-CoV-2 spike protein

**Figure 1.**
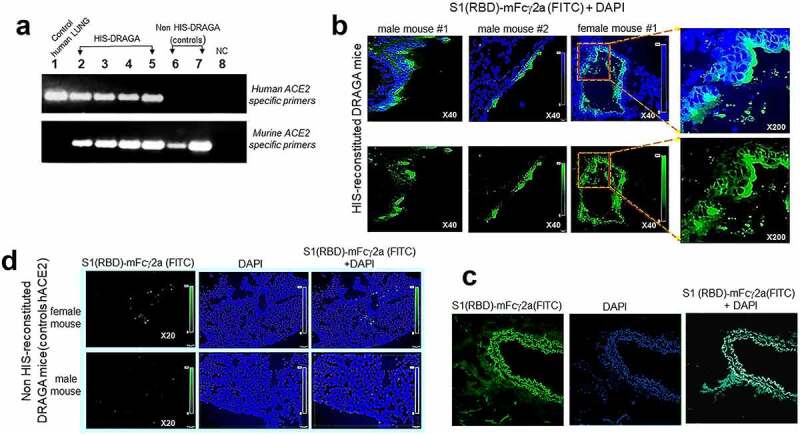
Human ACE2 detection in the lungs of HIS-DRAGA mice.

Human *ACE2* mRNA expression was detected in the lungs of non-infected, HIS-reconstituted DRAGA mice, but not in the control non-HIS reconstituted DRAGA mice (Figure 1a). Nucleotide sequencing of PCR amplicons confirmed the identity of hACE2 mRNA in HIS-DRAGA lungs (Figure S2 in supplementary data). To visualize binding of S1 (RBD) to human lung epi/endothelia, immunofluorescence microscopy was carried out on lung sections probed with S1 (RBD)-mFcγ2a. Digitized images revealed the S1 (RBD) bound to human lung epi/endothelial cells (Figure 1b) as well as to human lung sections (Figure 1c). Very weak S1 (RBD)-mFcγ2a staining was detected in lung sections from non-HIS reconstituted DRAGA mice (Figure 1d). The presence of hACE2 protein in the HIS-DRAGA lungs was further confirmed by staining with a mouse anti-hACE2 specific antibody and revealed by the same goat anti-mouse IgG-FITC conjugate as used to reveal bound S1 (RBD)-mFcγ2a (Figure S3 in supplementary data).

**(a)** Amplified RT-PCR products using hACE2 primers **(upper panel)** and mACE2 primers **(lower panel)** on RNA samples extracted from human lung (*lane 1*), from lungs of four non-infected HIS-DRAGA mice (*lanes 2–5*), and from lungs of 2 non-HIS-reconstituted DRAGA mice (*lanes 6–7*). NC, negative RT-PCR control (*lane 8*). **(b)** hACE2 protein expression on alveolar and bronchiolar epithelia as indicated by binding of S1 (RBD)-mFcγ2a recombinant chimeric protein revealed by a goat anti-mouse IgG-FITC conjugate in lung sections from two HIS-DRAGA male mice and one HIS-DRAGA female mouse. Lower panels show S1 (RBD)-FITC staining, and upper panels show S1 (RBD)-FITC and DAPI (nuclei) co-staining. Shown are enlargements of alveolar epithelia (right panels). (**c**) Lung section from human lung stained as in *panel* b. (**d**) Lung sections from two non-HIS reconstituted DRAGA mice stained as in *panel b.*

**Figure 2.**
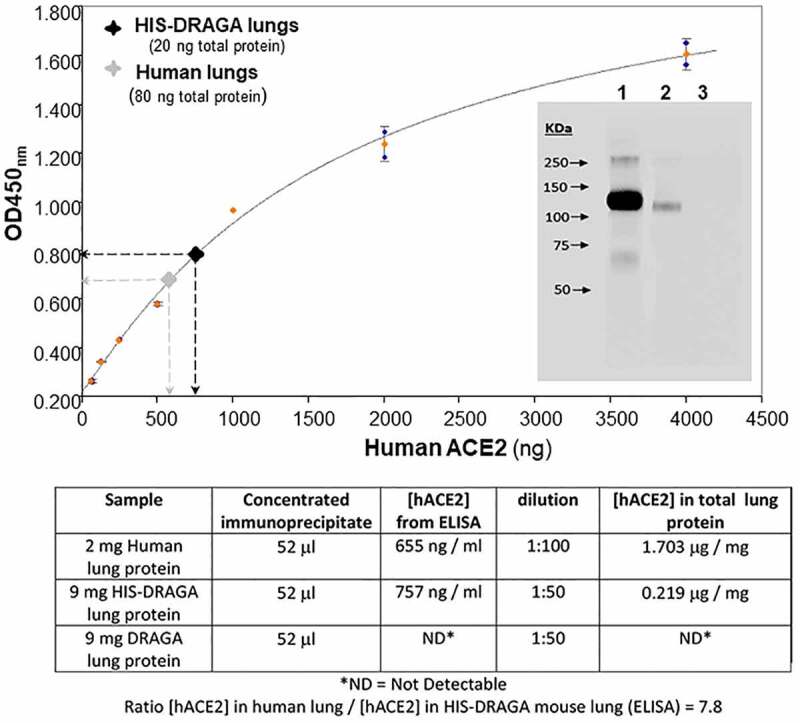
Quantification of hACE2 protein in HIS-DRAGA lungs.

To compare the amount of hACE2 protein in HIS-DRAGA lungs and human lungs, the recombinant SARS-CoV-2 S1 (RBD)-mouse Fcγ2a chimeric protein (S1 (RBD)-mFcγ2a) and magnetic beads coated with rat anti-mouse IgG2a were used to immunoprecipitate hACE2 from a pool of HIS-DRAGA lung homogenates (n = 10), non-HIS reconstituted DRAGA mice (n = 10) and a commercial human lung homogenate. Quantitative ELISA measurements indicated that hACE2 protein was 7.8 times less abundant in HIS-DRAGA mouse lungs than in human lungs, while no hACE2 was detected in the immunoprecipitates from non-HIS reconstituted DRAGA lungs (Figure 2). Immunoblot analysis revealed identical molecular weight of hACE2 protein expressed in HIS-DRAGA lungs and in human lungs (Figure 2, upper panel insert).

Lung lysates were immunoprecipitated using S1 (RBD)-mFcγ2a protein + rat anti-mouse IgG2a-magnetic beads, and eluted hACE2 levels were quantified by ELISA (upper panel), as described in Materials and Methods. Inserted image shows immunoblot analysis of hACE2 immunoprecipitated from human lungs (*lane 1*), hACE2 immunoprecipitated from HIS-DRAGA mouse lungs (*lane 2*), and the absence of hACE2 in the lung immunoprecipitate from lungs of a non-HIS-reconstituted DRAGA mice (*lane 3*). Lower panel provides the data used to calculate the amount of hACE2 immunoprecipitated from human lungs as compared to HIS-DRAGA mouse lungs.

**Figure 3.**
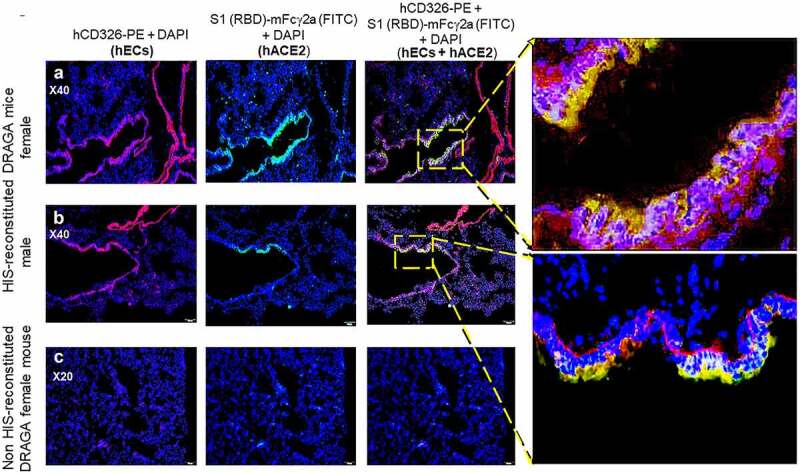
Co-Localization of hACE2 with hCD326 on alveolar human ECs in HIS-DRAGA lungs.

The human (h)CD326-specific marker for human lung endothelial cells (hECs) was also expressed on reconstituted human lung cells of HIS-DRAGA mice, as shown by co-staining with anti-hCD326-PE and S1 (RBD)-mFcγ2a that revealed co-localization of hACE2 with hCD326-expressing hECs (Figure 3, *upper* and *middle panels*), but the presence of hECs was not detected in non-HIS reconstituted DRAGA lungs (Figure 3, *lower panels*). Likewise, the upper respiratory airway (trachea) of HIS-DRAGA mice showed reconstitution of human CD326^+^ epithelial cells expressing hACE2 (Figure S4 in supplementary data). Microscopic examination of slides from all HIS-reconstituted DRAGA mice showed exclusive co-localization of hACE2 and CD326 marker. The results revealed that DRAGA mice infused with CD34^+^ HSCs reconstitute human ECs expressing hACE2 and hCD326^+^ in their lungs and upper respiratory airway.

(**a&b**) Co-localization of hACE2 protein with alveolar hCD326^+^ ECs revealed by co-staining with S1 (RBD)-mFcγ2a + goat anti-mouse IgG-FITC and anti-hCD326-PE in a representative HIS-DRAGA female and male mouse (upper panels). Shown are enlargements of CD326^+^ hECs co-localized with hACE2 on the alveolar epithelia. (**c**) Low background staining indicating lack of hCD326^+^ ECs and a lack of hACE2 expression lung section from a representative non-HIS-reconstituted DRAGA mouse.

### HIS-DRAGA mice sustain SARS-CoV-2 infection

**Figure 4.**
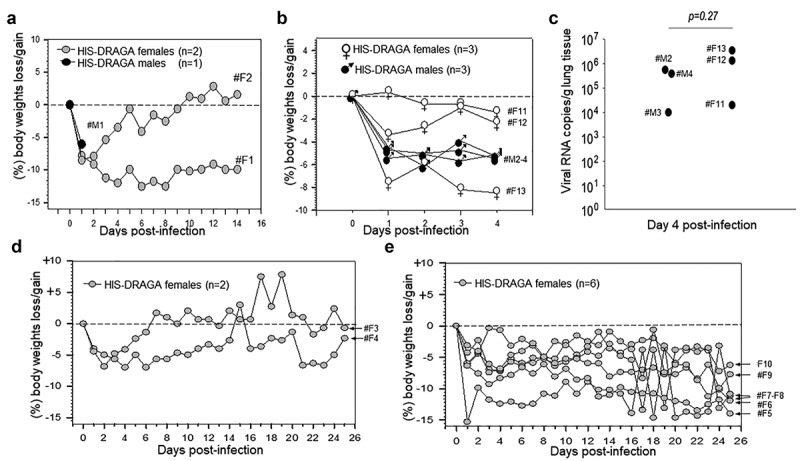
Dynamics of body weight changes following SARS-CoV-2 infection of HIS-DRAGA mice.

As a pilot experiment to determine whether HIS-DRAGA mice expressing hACE2 in lungs and upper respiratory tract can be infected with SARS-CoV-2 virus, three HIS-DRAGA mice were infused intranasally (i.n.) with relatively high doses of virus in 50 μl saline, 25 μl per nostril: 10^4^ pfu/mouse (male #M1 + female #F1) and 2.8x10^3^ pfu (female #F2). While the male mouse succumbed 24 h after infection, both female mice sustained the infection until the experimental endpoint of 14 days post-infection (dpi) (Figure 4a). Infected mice quickly showed an abrupt loss in body weight (likely due to severe dehydration), ruffed fur, hunched back, and reduced mobility starting at 1 dpi. Mouse #F2 regained its original weight and mobility by 9 dpi, while mouse #F1 was still 10% below its original weight and no amelioration of the clinical condition at 14 dpi. The results showing early weight loss in DRAGA mice at 1 dpi are in agreement with studies in SARS-CoV-2 infected human ACE2 transgenic mice, which also showed 5–10% body weight loss at 1 dpi and attributed to severe dehydration^52,53^

(**a**) Daily monitoring for body weights of HIS-DRAGA male mouse #M1 (dark circles) infected with SARS-CoV-2 virus (10^4^ pfu) and 2 HIS-DRAGA female mice #F1 and #F2 (gray circles) infected with 10^4^ pfu and 2.8x10^3^ pfu, respectively (pilot infection experiment). (**b**) Daily monitoring for body weights of 3 HIS-DRAGA female mice (#F11 - #F13, open circles) and three male mice (#M2 - #M4, dark circles) infected with SARS-CoV-2 virus (10^3^ pfu/mouse). (**c**) Viral RNA copies in the lungs of mice from *panel* B measured as described in the Materials and Methods. (**d**) Daily monitoring of body weights of 2 HIS-DRAGA female mice (#F3 and #F4) that recovered from infection after challenge with SARS-CoV-2 virus (10^3^ pfu/mouse). (**e**) Daily monitoring of body weights of six HIS-DRAGA female mice (#F5 - #F10, 6/8 mice, 75%) that did not recover from infection after challenge with SARS-CoV-2 (10^3^ pfu/mouse).

In a larger second infection experiment, 11 female HIS-DRAGA mice (#F3-F13) and 3 HIS-DRAGA male mice (#M2–4) were challenged i.n. with a lower dose of SARS-CoV-2 (10^3^ pfu/mouse) in 50 μl saline, 25 μl per nostril, and their body mass and clinical condition were monitored daily. All mice started to show ruffed fur, hunched back, and reduced mobility at 1 dpi, most likely due to severe dehydration. Furthermore, to measure the virus load in the lungs of infected mice, three females and three males (#F11-F13 and #M2-M4) that had not recovered their initial body weight by day 4 post-infection (Figure 4b), were euthanized and their lungs analyzed by RT-qPCR. The number of viral RNA copies in the lungs of these mice were in the range of 10^4^-10^6^ (Figure 4c) with no significant differences between females and males (p = .27). The remaining eight female mice in this group lost 5–15% body weight and showed deteriorating clinical conditions (ruffed fur, hunched back) starting at 1 dpi, most likely due to severe dehydration. However, mice #F3 and #F4 recovered the initial body weights by 7 dpi and 25 dpi, respectively (Figure 4d), while the remaining 6 mice (#F5-F10, 6/8 mice, 75%) had not recovered their initial body weights by the 25 dpi experimental endpoint (Figure 4e). Although the kinetics of infection in DRAGA model may differ from those in COVID-19 patients, results demonstrated that most HIS-DRAGA mice (75%) can sustain infection with SARS-CoV-2 virus for at least 25 days. Control groups of HIS-DRAGA mice challenged i.n. with PBS as we previously reported showed no loss in body weight during a 21-day period of time^49,50^

### HIS-DRAGA mice infected with SARS-CoV-2 display human-like lung pathology

**Figure 5.**
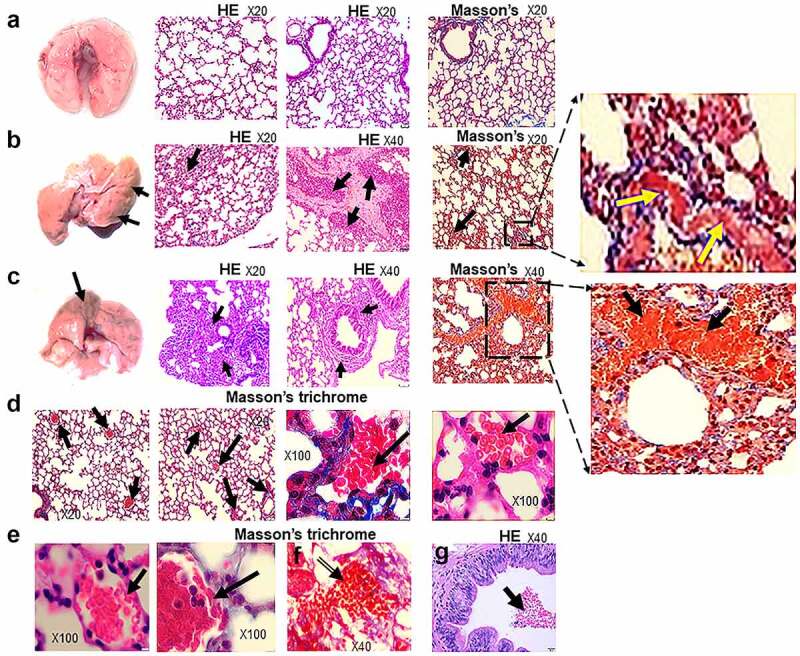
Lung pathology of SARS-CoV-2 infected HIS-DRAGA mice.

**Figure 6.**
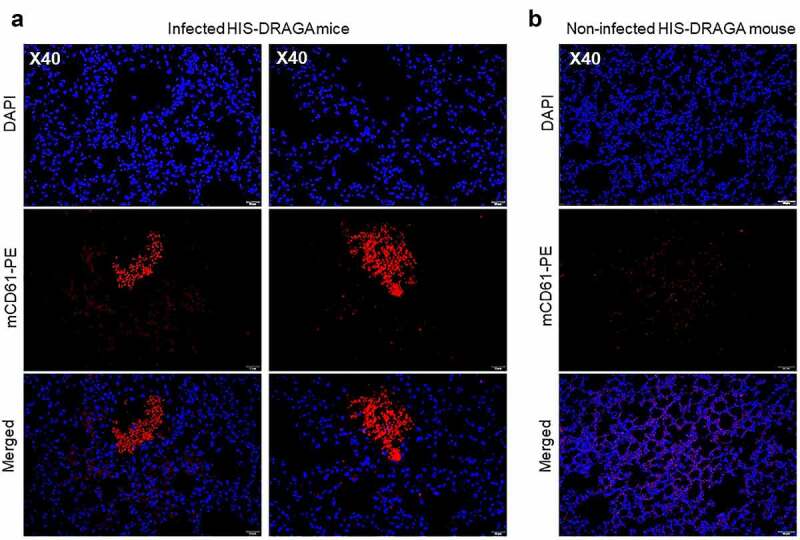
Intra-Alveolar microthrombi in SARS-CoV-2 infected DRAGA mice.

**Figure 7.**
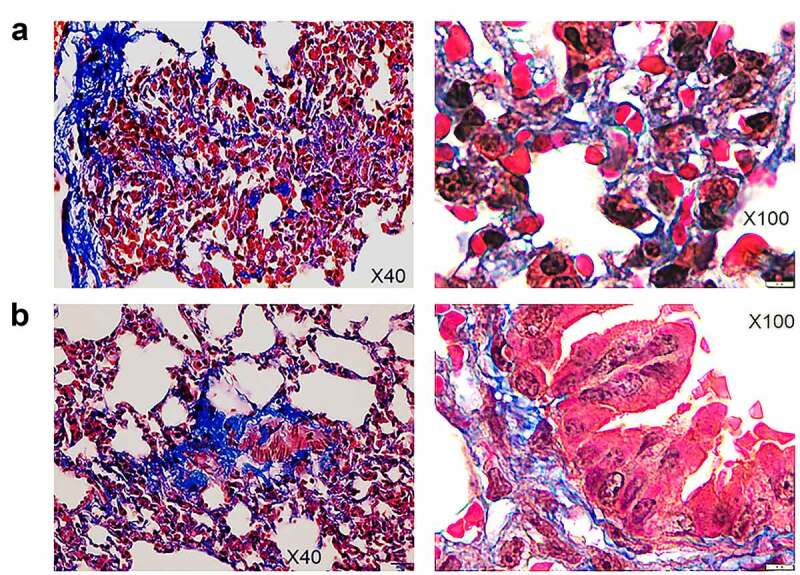
Pulmonary sequelae in SARS-Cov-2 infected HIS-DRAGA mice.

In contrast with non-infected HIS-DRAGA lungs analyzed in this study (Figure 5a), lungs from the HIS-DRAGA lungs infected with SARS-CoV-2 (10^3^ pfu/mouse) that had not recovered from infection at 25 dpi showed discolored areas, multiple interstitial and peri-bronchiolar infiltrates (Figure 5b,c). These mice also showed intra-arteriolar microthrombi (Figure 5d) with some adhering to endovasculature (Figure 5e), as well as interstitial (Figure 5f) and intra-bronchiolar blood clots (Figure 5g). The intra-alveolar microthrombi stained positive for the platelet marker CD61 (glycoprotein IIIa) (Figure 6). Some infected mice also showed excessive collagen deposition in the peri-alveolar infiltrated areas, indicating the presence of incipient pulmonary sequelae (Figure 7). Together, these results revealed that similar to humans with COVID-19, the HIS-DRAGA infected with SARS-CoV-2 virions developed diverse lung pathology.

(**a**) Images of lungs (*left panel*) and the H&E and Mason’s trichrome stained lung sections (*right panels*) from a representative non-infected HIS-DRAGA female mouse. (**b**) Image of lungs (*left panel*) and the H&E and Mason’s trichrome stained lung sections (*right panels*) from a representative HIS-DRAGA female mouse (#F5) infected with 10^3^ pfu of SARS-CoV-2, which did not recover its initial body mass by 25 dpi. Note the discoloration on the left lobe (arrows), parenchymal lymphocyte infiltration and hemorrhagic patches (arrows). Enlargement shows incipient peri-alveolar collagen deposition. (**c**) Image of lungs (*left panel*) and the H&E and Mason’s trichrome stained lung sections (*right panels*) from a representative HIS-DRAGA female mouse (#F6) infected with 10^3^ pfu of SARS-CoV-2, which had not recovered its initial body mass by 25 dpi. Note the discoloration on the left lobe (arrows) and heavy peri-bronchiolar infiltration with lymphocytes (arrows). Enlargement shows large hemorrhagic patches surrounding a major bronchiole (arrows). (**d**) Masson’s trichrome staining of lung sections from mouse #F5 in *panel b* showing intra-alveolar microthrombi (arrows) and collagen deposition (third left panel, blue color). Right panel shows an intra-alveolar blood clot. (**e**) Masson’s trichrome staining of lung sections (3 *left panels*) from mouse #F6 in *panel c* showing microthrombi adherent to the arteriole wall (two *left panels*, arrows) and an intra-alveolar hemorrhagic patch (*panel* f, arrow). H&E staining from the lungs of same mouse (#F6) showing a large intra-bronchiolar hemorrhagic patch (*panel* g, arrow).

(**a**) Lung sections from two HIS-DRAGA female mice (#F5 and #F6) infected with 10^3^ pfu that had not recovered from the infection by 25 dpi. Shown are large intra-alveolar, non-nucleated cell clusters staining positive for CD61, indicating the presence of platelet-rich microthrombi. (**b**) Representative lung section from a non-infected HIS-DRAGA female mouse; the lung parenchyma of this mouse showed no evidence of CD61^+^ -positive clusters indicating platelet-rich thrombi.

Masson’s trichrome staining of lung sections from female mice #F5 (*panel* a) and #F6 (*panel* b) which did not recover their initial body weights by 25 days post-infection with SARS-CoV-2 (10^3^ pfu/mouse). Shown are collagen fibers (blue color) in the peri-alveolar and intra-alveolar infiltrated areas (*left panels*) and around major bronchioles.

### Pulmonary infiltrates in SARS-CoV-2 infected HIS-DRAGA mice contain human lymphocytes and lung-resident human T cells

**Figure 8.**
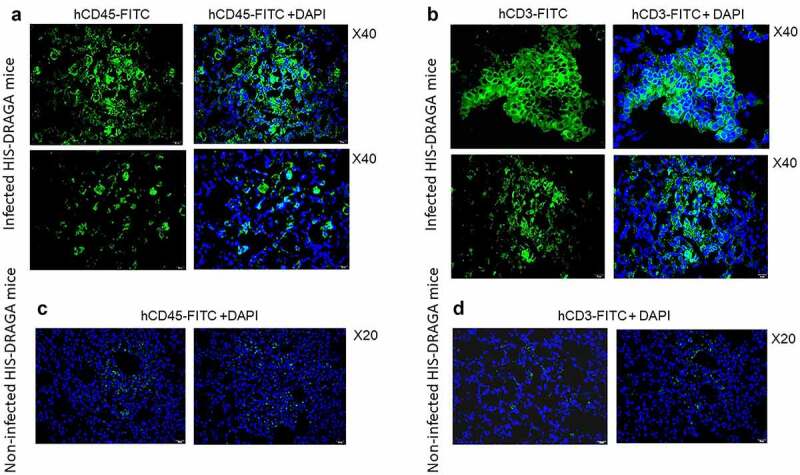
Infiltrating human lymphocytes in the lungs of SARS-CoV-2 infected HIS-DRAGA mice.

**Figure 9.**
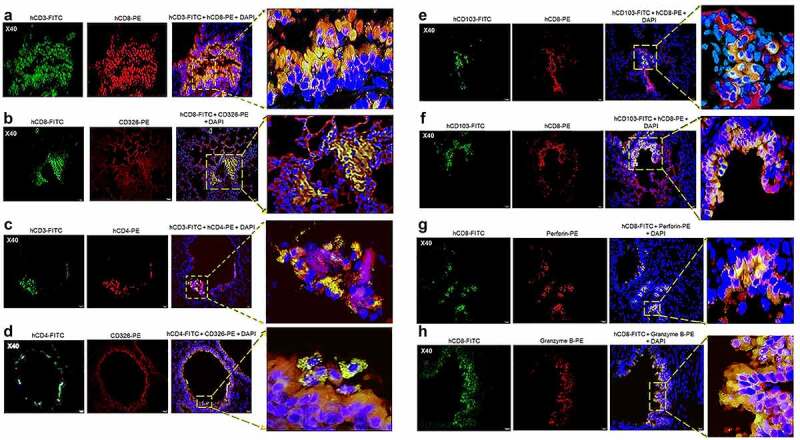
Human CD8^+^ T cell residency and cytotoxicity in the hCD326^+^ lung epithelial niches of a SARS-CoV-2-infected HIS-DRAGA mouse.

Lung infiltrates visualized in mice infected with a low dose of SARS-CoV-2 virions (10^3^ pfu/mouse) stained positive for hCD45, indicating the presence of human lymphocytes (Figure 8a) and hCD3 (Figure 8b), indicating the abundance of human T cells. Infiltrating hCD45^+^ lymphocytes and hCD3^+^ T cells were less abundant in the lungs of non-infected HIS-DRAGA mice, mostly present in the alveolar vasculature rather than in alveolar air space (Figure 8c, d). Among the hCD3 T cell infiltrates, some hCD8^+^ and hCD4^+^ T cell subsets co-localized with the alveolar CD326^+^ hECs, and some were egressing into the alveolar air space (Figure 9a-d). The CD326^+^ hCD8 T cell lung infiltrates were organized in clusters in two mice (#F3 and #F4) that had recovered from the infection by 25 dpi (Figure 9e-h). Interestingly, the hCD3 T cell clusters in the lung infiltrates from these same two mice stained positive for hCD103, a marker for T cell residency (Figure 9e,f), as widely described in humans and Rhesus monkeys infected with upper respiratory viruses including influenza type A viruses.^54,55^ To determine if resident hCD8 T cells clustered in the lung niches of these mice were potentially cytotoxic, lung sections were stained for perforin and Granzyme B. Among the hCD8^+^ T cells sequestered in alveolar hEC niches, some stained positive for perforin and granzyme B, suggesting anti-viral cytotoxicity (Figure 9g,h). Fewer hCD3 T cells stained positive for perforin and granzyme B, and very few hCD3 T cells stained positive for hCD103 in the mice that had not recovered from infection by 25 dpi (Figure S5 in supplementary data). Microscopic screening of lung sections for individual mice from the largest infection group revealed three times higher number of CD8^+^CD103^+^ T cell patches on average in those recovering from infection (#F3, 9 patches per 1/section and #F4, 10 patches/section) than in those which did not fully recover their initial body mass (#-F5-F10, 1 to 4 patches/section) at 25 days post-infection. For the time being, the fluorescence distribution and single-cell fluorescence intensity of positive cells have not been carried out.

Lung sections from HIS-DRAGA female mice #F4 (*upper panels* in **a** & **b**) and #F3 (*lower panels* in **a** & **b**) that had recovered from SARS-CoV-2 infection(10^3^ pfu/mouse) by 25 dpi stained for hCD45 and hCD3. Of note, infiltrating hCD45^+^ lymphocytes and hCD3^+^ T cells in the infected mice were more abundant and organized in clusters than those in non-infected mice that were dispersed through parenchyma (*panels* & d).

Lung sections from a representative HIS-DRAGA female mouse (#F3) that had recovered its initial body weight 7 days post-infection with SARS-CoV-2 (10^3^ pfu). (**a-d**) Co-localization of hCD3^+^ CD4^+^ and CD8^+^ T cell subsets with the hCD326^+^ epithelia marker (mouse #F3). Enlargements show areas of CD4^+^ and CD8^+^ T cell clusters sequestered in the lung CD326^+^ epithelia, with some CD8^+^ T cells egressing into the alveolar air space. (**e&f**) hCD8^+^ T-resident cell clusters sequestered in lung epithelia of mouse #F3 expressing the hCD103 marker of lung residency. (**g&h**) hCD8^+^ T cell resident clusters in bronchiolar epithelia of mouse #F3 staining positive for perforin and granzyme B. Similar findings were also present in the lungs of HIS-DRAGA female mouse (#F4) that had recovered its initial body weight by 25 days post-infection.

Together, these analyses revealed the presence of human lymphocyte infiltrates in SARS-CoV-2 infected lungs of HIS-DRAGA mice, and that human lung-resident CD103^+^ hCD8 T cells secreting perforin and granzyme B were more abundant in the lungs of mice that recovered from infection, suggesting potential anti-viral cytotoxicity.

### HIS-DRAGA mice recovering from SARS-CoV-2 infection mounted IgG antibody response to the viral proteins

**Figure 10.**
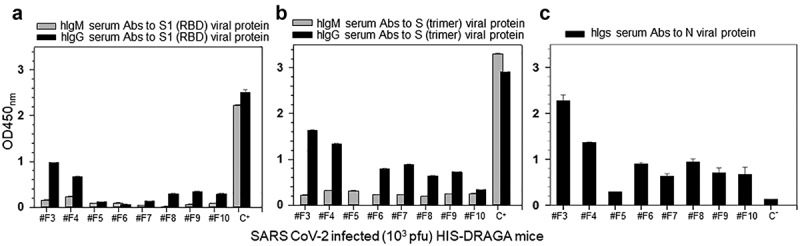
Human IgM and IgG serum titers to SARS-CoV-2 viral proteins in infected HIS-DRAGA mice.

Infected mice that recovered their initial body weights by 25 dpi (#F3 and #F4) showed the highest human antibody responses to the SARS-CoV-2 S1(RBD), S(trimer) and N proteins, whereas those with over 5% loss in body weight that did not recover from infection (#F5-F10) had lower serum antibody responses to the viral proteins (Figure 10a). The same mice that mounted the highest IgG response to S1 (RBD) also mounted a higher IgG response to the S trimer and N viral proteins (Figure 10b,c). Overall, the IgM antibody responses to the S1 (RBD) and stable S trimer proteins were low in all mice at the 25 dpi experimental end-point, most likely due to immunoglobulin switch class, as we have recently described in DRAGA mice infected with influenza.^27^ Though only two out of eight mice were able to recover body weight by 25 dpi, statistical analyses indicated significant correlations between body weights and human IgG Abs to S1(RBD) (Spearman’s rs = (+).9047, P = .002) and with total Igs to N protein (rs = (+). 71,429, P = .046) but not with human IgG Abs to S trimer (rs = (+).2857, P = .492) or with IgM Abs to S1(RBD) (rs = (+).3809, P = .351) or S(trimer) (rs = (-).023, P = .955). There were no detectable IgM or IgG serum antibodies in HIS-DRAGA mice infected i.n with a sub-lethal dose of influenza PR8 virus (4x10^−4^ EID_50_/mL/mouse in 20 μL PBS)^49^as measured by ELISA at day 21 post-infection (Figure S6 in supplementary data). Overall, these results indicated that SARS-CoV-2 infected HIS-DRAGA mice recovering from infection mounted higher human anti-viral responses than those unable to recover from infection, suggesting a role of human anti-viral antibodies for faster recovery from infection. There was, however, no significant correlation between the SARS-CoV-2 human antibody responses elicited by DRAGA mice at 25 dpi and the percentages of human B cells or human T cells in peripheral blood of DRAGA mice prior to infection (Spearman correlation p > .05).

Shown are the hIgM and hIgG serum titers to SARS-CoV-2 S1 (RBD) protein (**a**), stable S trimer (**b**) and total serum antibody titers to Nucleocapsid protein (**c**) quantified by ELISA at 25 days post-challenge with SARS-CoV-2 (10^3^ pfu/mouse). OD450_nm_ values of IgM and IgG antibodies specific for S protein provided in the ELISA kit are indicated (control C+). Control for serum Abs to Nucleocapsid protein (C-) was obtained from a pool of non-infected HIS-DRAGA mice. Shown are the OD450_nm_ values of anti-viral antibodies corrected by subtracting the OD570_nm_ values in each well and the signal-to-noise background (ranging from 0.045 to 0.067) from sera of individual mouse prior to infection. Standard deviations (±SD) from two repeated measurements for each serum sample in duplicate wells were determined at 99% interval of confidence using SigmaPlot v.14 software.

## Discussion

In this work, HIS-humanized DRAGA mice expressing functional human immune system were found to reconstitute human epi/endothelial cells in lungs and upper respiratory airway expressing hACE2, the primary receptor in humans for SARS-CoV-2. They also reconstitute various hematopoietic cell-derived human cells, including endothelial cells (EDs) in the liver^45^and epithelial cells (ECs) in the lungs.^50^ The pluripotency of umbilical cord blood CD34^+^ stem cells to differentiate into non-hematopoietic cells, such as hepatocytes, neural, lung, and gastric epithelial cells in humans and animal models has been widely demonstrated.^56–61^ Also, umbilical cord blood-derived CD34^+^ HSC were shown to proficiently differentiate into alveolar lung ECs in mice,^62^ which is in agreement with results from our study in DRAGA mice showing reconstitution of human alveolar lung ECs.

HIS-DRAGA mice sustained infection via the intranasal route with non-mouse-adapted SARS-CoV-2 for at least 25 days. Due to limited number of female and male mice used in this study, the gender difference with respect to the outcomes of SARS-CoV-2 infection cannot be ruled out. A larger number of female and male DRAGA mice will be required to determine statistically relevant differences on the outcomes of infection. Several molecular and cellular mechanisms have been suggested for higher resistance, less severity, and faster recovery of SARS-CoV-1 and CoV-2 in women than in men^63–66^with estrogens at the top of the list. Thus, in a recent Italian study 63.9% of the total fatalities were men.^67^ Another study found that due to the protective effect of estrogen, from more than 160,000 COVID-19 patients, 74% of them were men and only 26% women.^68^ Also, studies of Garg et al. highlighted the protective role of estrogen against COVID-19 by finding a 12.8% mortality rate in postmenopausal compared with 8.6% in premenopausal women.^69^ Further investigations in DRAGA mice may determine which of the molecular and cellular mechanisms responsible for faster recovery from infection are operational, and whether similar beneficial effects of estrogens like in COVID-19 patients may occur in these mice.

Future research will also require assessments of whether infected HIS-DRAGA mice can naturally transmit SARS-CoV-2, as in our recent demonstration of natural influenza transmission between co-caged, infected, and non-infected HIS-DRAGA mice.^49^ Another compelling priority for future research that is ongoing in our labs is to investigate the outcome of infection with emerging SARS-CoV-2 variants of concern (VOC), evaluate immunopathological responses and characterize the human antiviral antibodies generated by these mice in relation to the magnitude of infectious dose and severity of disease. A case-report for the first COVID-19 patients showed that the virus was undetectable by day 14 after diagnosis,^70^ while studies on large cohorts of patients at various stages of disease showed that the duration of viable virus is relatively short-lived (i.e.,17 days).^71,72^ We thus measured the viral load in DRAGA lungs at day 4 post-infection. To find out any correlations with human studies, a larger number of DRAGA mice will be required to monitor the viral kinetics during the infection.

As described in COVID-19 patients,^1–4^ SARS-CoV-2 infected HIS-DRAGA mice developed mild to severe lung immunopathology including T cell infiltrates, alveolar damage, intra-alveolar and intra-arteriolar microthrombi, bronchiolar blood clots, and collagen deposition. Furthermore, as in autopsy samples from COVID-19 patients,^73–75^ the lung infiltrates of infected HIS-DRAGA mice were interstitial and clustered around terminal bronchioles. These infiltrates contained resident (CD103^+^) human T cells, particularly hCD8^+^ T cells clustered in hCD326^+^ lung epithelial niches, and some were visualized egressing in the alveolar air space. Many resident hCD8 T cells stained positive for granzyme B and perforin, suggesting potential anti-viral cytotoxicity. Lung-resident CD8^+^ T cells have been detected and characterized in mice, monkeys, and humans.^76,77^ Recently, we have also described the presence of lung-resident CD103^+^ hCD8^+^ T cells in the lungs of influenza-infected HIS-DRAGA mice.^49^ Lung-resident CD8 T cells are positioned to act at the frontline of lung epithelial mucosa after a primary viral respiratory infection to provide rapid and efficient cross-protection against subsequent exposures to respiratory viruses.^76,77^ Microscopic screening of the largest group of infected mice revealed three times higher number of CD8 lung-resident T cell patches on average in those recovered from infection than in those not fully recovered at 25 days post-infection. Further investigations will be required to determine whether human lung-resident CD8 T cells can protect against COVID-19, which can be addressed in HIS-DRAGA mouse model by adoptive cell transfer experiments.

SARS-CoV-2 infected HIS-DRAGA mice were also able to mount human antibody responses to S1 (RBD), S-trimer and Nucleocapsid viral proteins, with those able to recover from infection having the highest IgG titers. Recent studies in animal models and humans have suggested critical roles for both antibodies^78–80^ and T cells^81,82^ in protection against COVID-19. Such findings in both humans and HIS-DRAGA mice raised a question that needs to be further addressed: are antibodies, lung-resident CD8^+^ T cells, or both critical for efficient protection against COVID-19. While investigation of the dynamics of antibody titers versus virus clearance in COVID-19 patients are feasible in humans, exploring the dynamics of cellular subsets in infected human subjects is not, as studies of human lungs are restricted to analyses of single biopsy/resection or post-autopsy samples. These challenging investigations can be addressed in the HIS-DRAGA mouse model for COVID-19.

Thrombophilia, including microthrombi in the lung, sometimes termed “immunothrombi” due to their association with the hyperinflammatory response, are a feature of severely infected COVID-19 patients.^6–9^ Histologic analysis of pulmonary vessels from COVID-19 autopsy samples showed widespread thrombosis with microangiopathy.^6–9^ In lungs, new vessel growth occurs predominantly through a mechanism of intussusceptive angiogenesis.^83^ In addition, occlusion of alveolar capillaries described in patients with severe COVID-19^10^ and adherence of microthrombi to the vascular endothelium suggest a distinctive angiocentric feature.^10^ The lungs of infected HIS-DRAGA mice had clusters of non-nucleated cells staining positive for CD61, consistent with the presence of platelets microthrombi in the alveolar air space. Infected mice unable to recover from SARS-Cov-2 infection showed hemorrhagic patches in the lungs, mimicking those described in autopsy samples from humans exiting COVID-19.^73,75^

Pulmonary fibrosis, also known as sequelae, indicates tissue scarring during healing that occurs by deposition of collagen in heavily infiltrated areas, as revealed in the lungs of patients recovering from severe respiratory infections. Radiographic and autopsy data have identified pulmonary fibrosis not only in COVID-19, but also in SARS-CoV-1 and MERS.^84^ It has been suggested that collagen deposition in severe lung injury by SARS viruses relies primarily on trafficking circulating fibrocytes to the lungs^85^ and increased signaling through the epidermal growth factor receptor.^86^ Masson’s trichrome staining of lung sections from SARS-CoV-2-infected HIS-DRAGA mice that sustained low-dose infection for up to 25 days and did not recover from infection revealed incipient collagen depositions in heavily infiltrated peri-alveolar and peri-bronchiolar areas. Of note, the course of infection as well as SARS-CoV-2 induced pathological events and T cell and antibody responses to the infection did not strictly correlate with the frequency of human T and B cells in the blood stream of HIS-DRAGA mice at the time of infection, suggesting that the adaptive immune responses are not solely involved in protection against COVID-19. Recently, a human-immune-system mouse model of COVID-19 using adeno-associated virus to deliver transiently the hACE2 to the mouse lungs, has been reported.^87^ The HIS-DRAGA mouse naturally reconstitute human cells expressing hACE2 not only in the lungs but also in brain, liver, kidney, and small intestine (Vir et al., manuscript in preparation)

In summary, the results of this study showed first that pluripotent human CD34^+^ HSC from the umbilical cord blood differentiated not only into functional human T and B cells, but also into human ECs and EDs expressing hACE2 in the lungs of HIS-DRAGA mice. Secondly, HIS-DRAGA mice sustained infection with SARS-CoV-2 for at least 25 days, exhibited clinical symptoms, and some recovered from infection and mounted an IgG anti-viral response. Thirdly, evaluations of their lungs revealed human-like pathological events including parenchymal and peripheral lung hCD3^+^ T cell infiltrates, intra-alveolar and intra-arteriolar microthrombi adherent to the endovasculature, intra-bronchiolar blood clots, and incipient pulmonary sequelae. The HIS-humanized DRAGA *in vivo* surrogate mouse model may offer compelling advantages for studying the immunopathological mechanisms of COVID-19. Notably, the ability to analyze physiological responses and harvest tissues at specific time points following infection and subsequent viral challenges in this animal model can provide vitally important mechanistic information that is not accessible in humans, since harvesting lung samples from patients with an ongoing infection is not feasible. This HIS-humanized DRAGA mouse model may also prove useful for efficient preclinical testing of both safety and efficacy of vaccines and potential therapeutics for human COVID-19.

*Limitations of this study*: As this work was primarily focused on establishing a HIS-humanized mouse model for SARS-CoV-2 infection and characterizing the lung immunopathology, there are few limitations, that is, viral replication in the lungs and other tissues has not been analyzed later than 4 days post-infection, and assessment of a possible “cytokine storm” and virus-specific T cell responses have not been attempted yet. Follow-on studies are now in progress to further characterizing the immunopathology and dynamics of live virions by TCID50 in the lungs and other tissue organs of infected HIS-DRAGA mice with SARS-CoV-2 VOCs.

## Supplementary Material

Supplemental Material

## Data and animal availability statement

Additional datasets generated and/or analyzed during the current study are available from the corresponding authors on reasonable request. HIS-humanized DRAGA mice will be made available to the scientific community under US Cooperative Research and Development Agreements approved by the Naval Medical Research Center, Silver Spring, MD.
