# Supplementary figures and images for: Human-Immune-System (HIS) humanized mouse model (DRAGA: HLA-A2.HLA-DR4.Rag1KO.IL-2RγcKO.NOD) for COVID-19
Source: Hum Vaccin Immunother. 2022 Mar 29;18(5):2048622. doi: 10.1080/21645515.2022.2048622 (PMC9225593; doi:10.1080/21645515.2022.2048622)

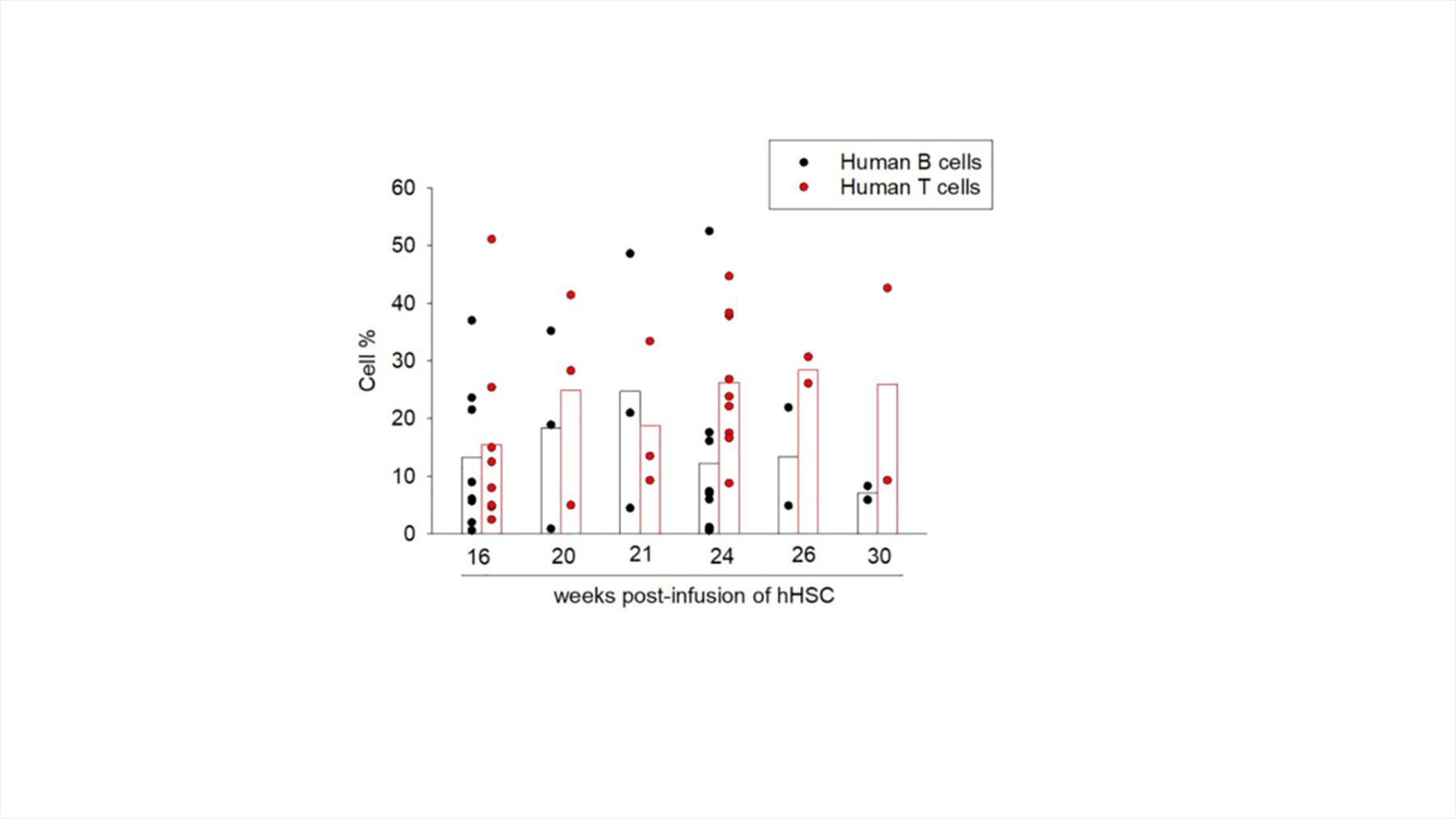

Supplement: Supplemental Material [file KHVI_A_2048622_SM2707.zip › Supplemental Material_2048622/Figure S1.tif]

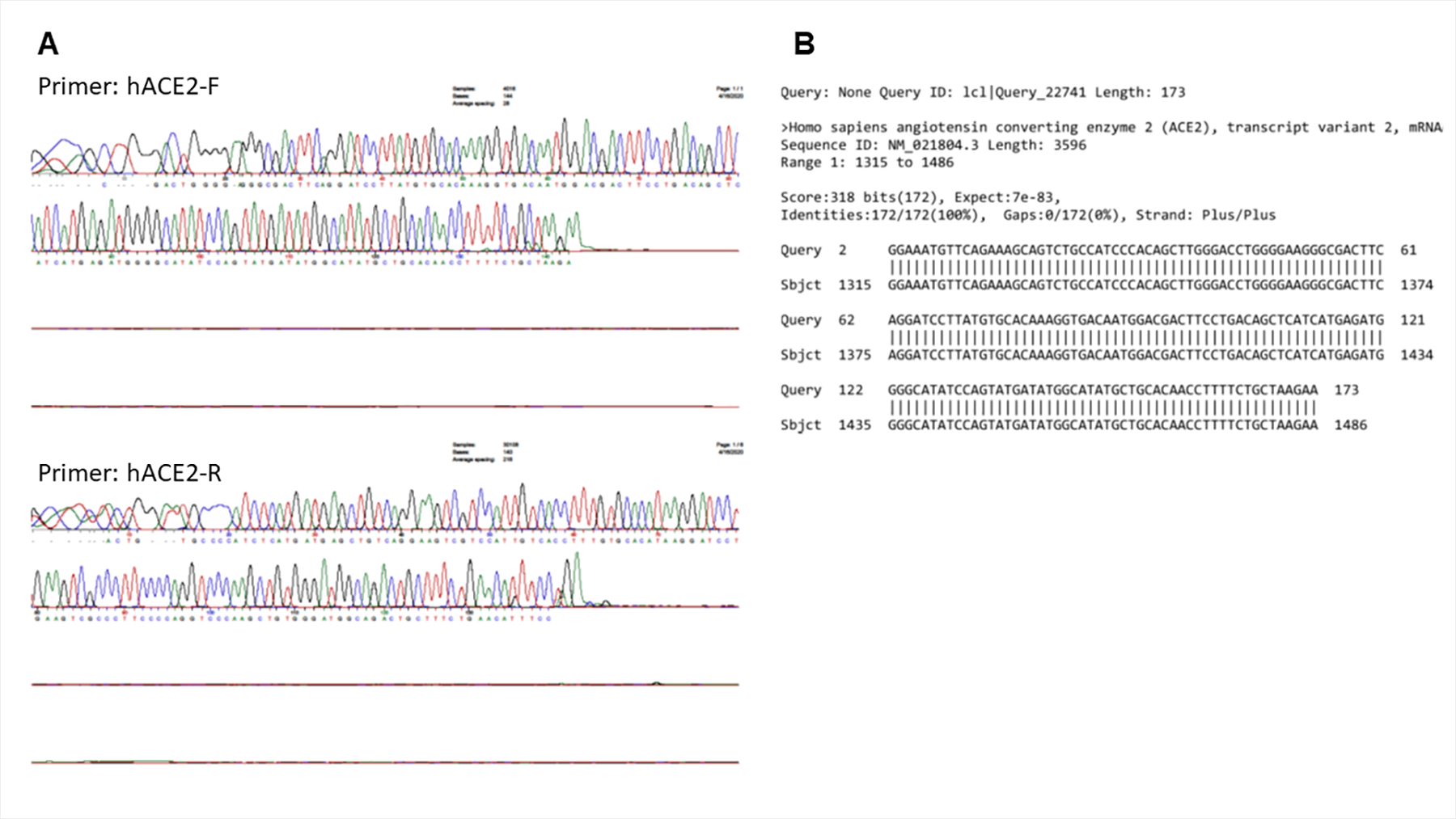

Supplement: Supplemental Material [file KHVI_A_2048622_SM2707.zip › Supplemental Material_2048622/Figure S2.tif]

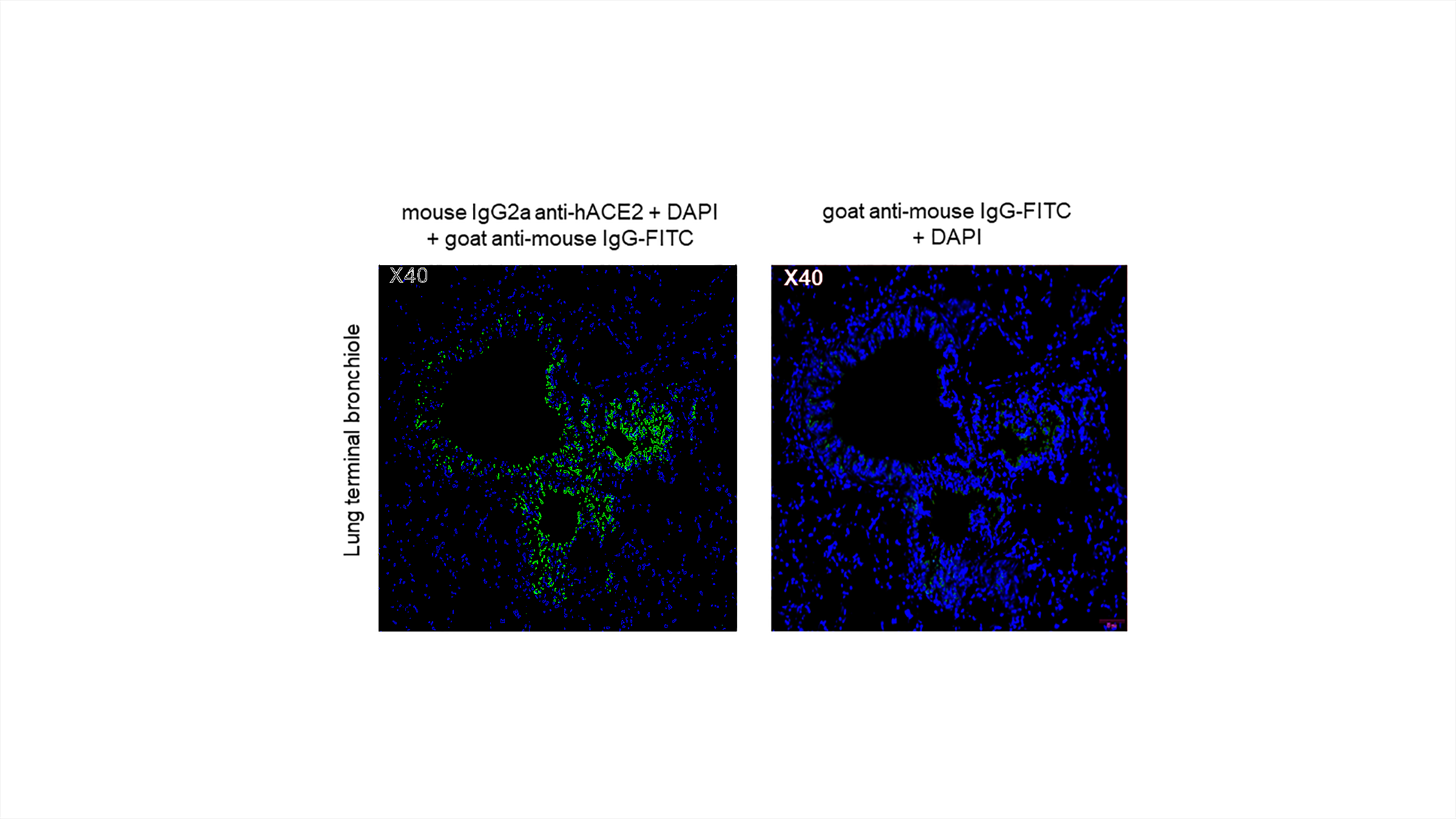

Supplement: Supplemental Material [file KHVI_A_2048622_SM2707.zip › Supplemental Material_2048622/Figure S3.tif]

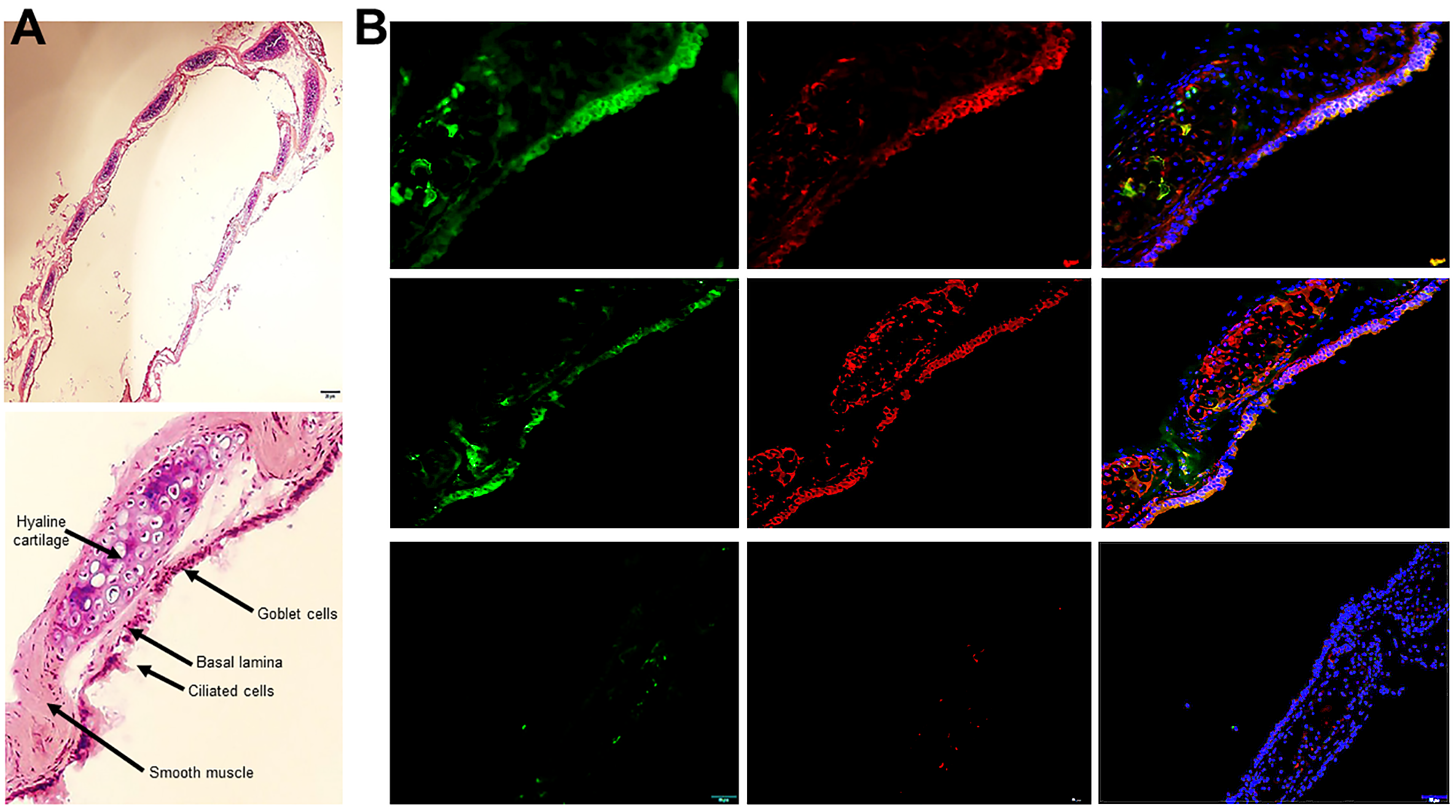

Supplement: Supplemental Material [file KHVI_A_2048622_SM2707.zip › Supplemental Material_2048622/Figure S4-revised.tif]

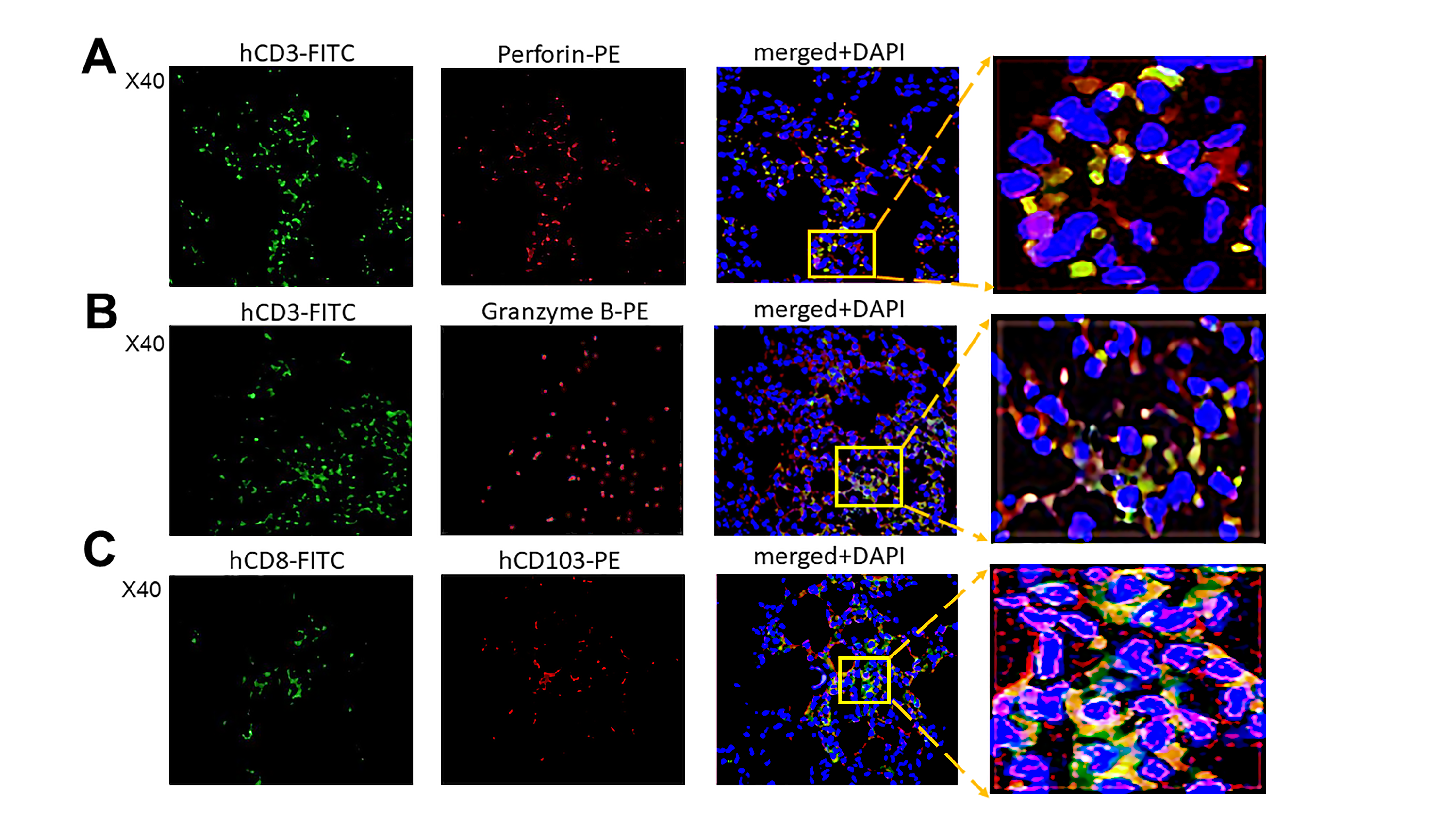

Supplement: Supplemental Material [file KHVI_A_2048622_SM2707.zip › Supplemental Material_2048622/Figure S5-revised.tif]

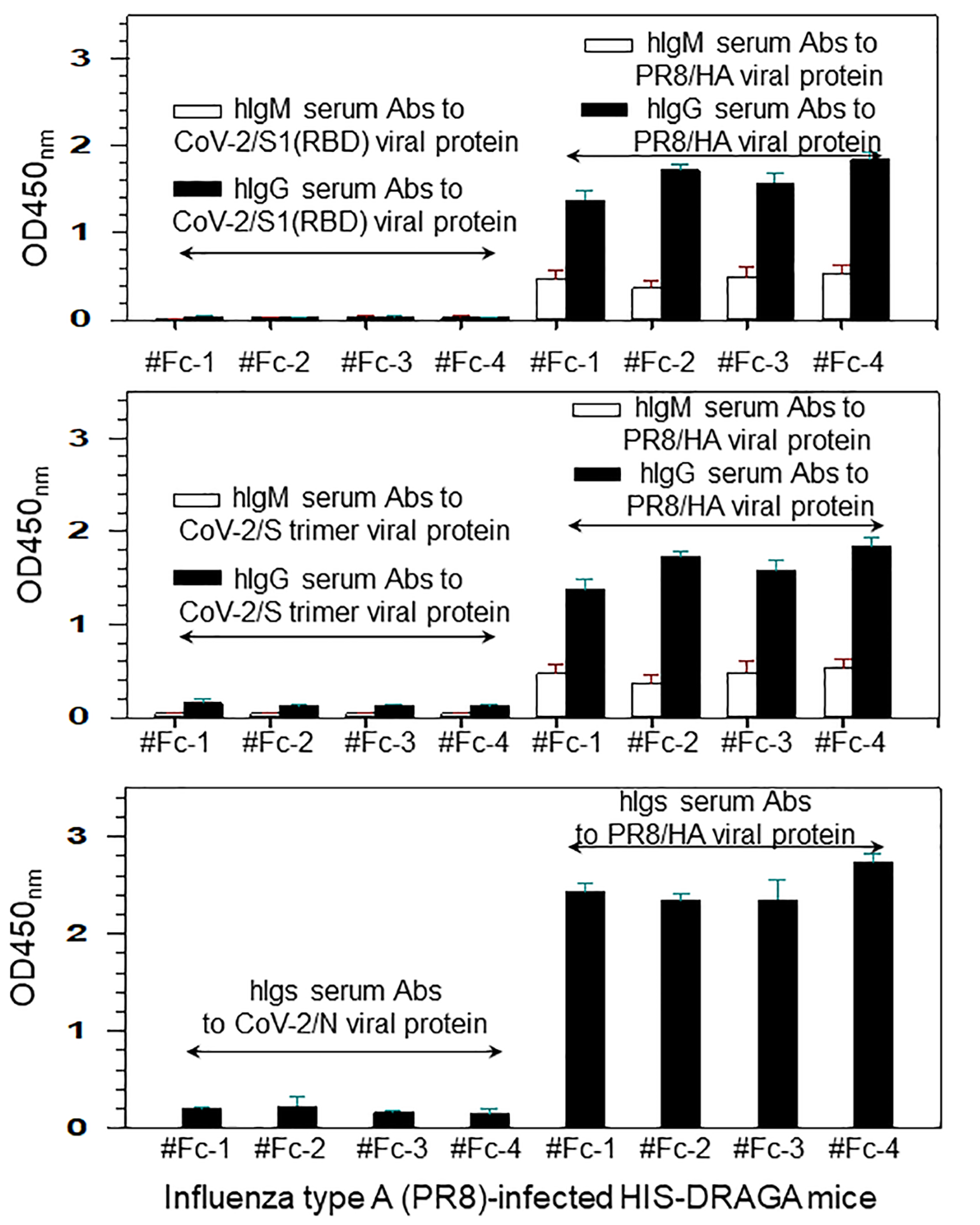

Supplement: Supplemental Material [file KHVI_A_2048622_SM2707.zip › Supplemental Material_2048622/Figure S6-revised.tif]
